# A Case of Cushing's Disease and a RET Pathogenic Variant: Exploring Possible Rare Associations

**DOI:** 10.7759/cureus.71058

**Published:** 2024-10-08

**Authors:** Guilherme Vaz de Assunção, Ana Miguel Capela, Liliana Fonseca, Cláudia Falcão Reis, Cláudia Amaral

**Affiliations:** 1 Endocrinology, Diabetes, and Metabolism, Centro Hospitalar Universitário de Santo António, Unidade Local de Saúde de Santo António, Porto, PRT; 2 Medical Genetics, Genetics and Pathology, Centro Hospitalar Universitário de Santo António, Unidade Local de Saúde de Santo António, Porto, PRT; 3 Medical Genetics, Life and Health Sciences Research Institute (ICVS) 3B’s Research Group, PT Government Associate Laboratory, Braga, PRT; 4 Medical Genetics, Unit for Multidisciplinary Research in Biomedicine, Abel Salazar Biomedical Sciences Institute, Porto University, Porto, PRT; 5 Medical Genetics, Life and Health Sciences Research Institute (ICVS) University of Minho, Campus de Gualtar, Braga, PRT

**Keywords:** case report, cushing disease, endocrine familial disorders, men2, pathogenetics, ret gene variant

## Abstract

Cushing disease (CD), a rare endocrine disorder characterized by a pituitary adenoma that secretes excess adrenocorticotropic hormone (ACTH), leads to overproduction of cortisol by the adrenal glands and, depending on severity and duration, manifests with a broad spectrum of clinical signs and symptoms, ranging from classical features to more common conditions seen in the general population. Discovery of molecular and pathogenic mechanisms related to the development of CD tumors has increased in recent years, almost two-thirds of the somatic variants cases have been linked to the USP8 gene, while very rare germline variants in MEN1 and AIP genes have been associated with pituitary adenomas. Variants affecting the RET proto-oncogene, which encodes a receptor tyrosine kinase involved in cell growth and differentiation, are implicated in the development of medullary thyroid carcinoma (MTC) and its hereditary form, multiple endocrine neoplasia type 2 (MEN2). This genetic syndrome is also associated with extra-thyroidal manifestations, such as pheochromocytoma, hyperparathyroidism, Hirschsprung's disease, mucosal neuromas, or cutaneous lichen amyloidosis. In both hereditary and sporadic forms of MTC, genetic testing is essential for promoting preventive strategies for first-degree relatives and facilitating early diagnosis.

We report a case of a 35-year-old male with a history of hypertension, bilateral carotid artery aneurysms, and intracranial fusiform dolichoectasia, with clinical manifestations suggestive of Cushing syndrome. Laboratory investigation confirmed ACTH-dependent hypercortisolism. However, magnetic resonance imaging did not reveal any pituitary tumor. A bilateral inferior petrosal sinus sampling confirmed the diagnosis of CD. The patient underwent successful transsphenoidal endoscopic surgery to remove the corticotropinoma, resulting in significant biochemical and clinical improvement. Due to the patient history of multiple vascular abnormalities and the suspicion of a possible genetic connective tissue disorder, comprehensive genetic testing with whole exome sequencing was performed, identifying a heterozygous pathogenic variant in RET (c.2410G>T p.{Val804Leu}). This variant has been previously associated with MEN2 manifestations and described as having a moderate risk for aggressive MTC and a low risk for pheochromocytoma and hyperparathyroidism. Genetic testing of available first-degree relatives for the RET variant was negative. To our knowledge, this is the third reported case of Cushing disease in a patient with a RET variant. This rare association can be due to coincidence, but we cannot exclude the possibility that the two conditions could share a common pathogenic mechanism. Although further research is needed to firmly establish a possible association, this case also highlights the necessity of exploring genetic backgrounds when patients present with clinical manifestations not readily explained by a single endocrine disorder. Investigating potential genetic associations is crucial since a positive genetic test allows for the testing of relatives, genetic counseling, and proper surveillance of individuals at risk.

## Introduction

Cushing syndrome (CS) encompasses all causes of hypercortisolism, whether endogenous or exogenous. Cushing disease (CD) is the most common form of endogenous CS, responsible for approximately 70% of cases [1,2]. CD specifically refers to the endogenous condition caused by an adrenocorticotropic hormone (ACTH)-secreting pituitary adenoma or corticotropinoma, with an estimated incidence of two to three cases per million individuals per year. Patients with CD often experience a poorer long-term quality of life compared to those with other forms of CS [1-3]. Depending on the severity and duration, the exposure to elevated cortisol levels can manifest with a broad spectrum of clinical signs, ranging from classic features - facial fullness and plethora, reddish-purple abdominal striae, proximal muscle weakness, and easy bruising - to more common conditions seen in the general population, including diabetes mellitus (DM), central obesity, depression or arterial hypertension [1]. The diagnostic approach begins with screening and confirmation of endogenous hypercortisolism, ruling out pseudo-Cushing syndrome, and determination of plasma adrenocorticotropic hormone (ACTH) levels [2,3]. Following the diagnosis of ACTH-dependent hypercortisolism, the next step is to determine the localization of the neoplasm with a pituitary magnetic resonance imaging (MRI) scan or, if necessary, through a bilateral inferior petrosal sinus sampling (BIPSS), allowing differentiation between a pituitary source and ectopic ACTH secretion [2]. Transsphenoidal surgery to remove the ACTH-secreting pituitary adenoma is the first-line treatment for CD. Remission is achieved in approximately 80% of patients with microadenomas and 60% of those with macroadenomas. In cases where surgery is not feasible or is unsuccessful, medical treatments, radiation therapy, or bilateral adrenalectomy can be used as second-line therapeutic options, depending on the individual.

Corticotropinomas are described to originate from pathogenetic pathways caused by either somatic or germline deleterious variants. Somatic gain of function variants in USP8 is the most frequent with CD, targeting the epidermal growth factor receptor (EGFR), stimulating the production of pro-opiomelanocortin (POMC), and subsequent secretion of ACTH. Somatic variants in TR4 and BRAF genes have also been associated with overexpression of POMC. More rarely, CD occurs in the context of familial syndromes. The AIP gene has been strongly associated with familial isolated pituitary adenoma (FIPA), and three reported cases have shown an association between CD and this genetic condition. Another germline variant associated with CD is the multiple endocrine neoplasia type 1 (MEN1). Its prevalence is also rare, and the variants occur in the MEN1 gene, with loss-of-function of the encoded protein menin, which acts as a tumor suppressor. Very few case reports mention CD with MEN2 or MEN4, although its definite association is yet to be established [4].

Deleterious variants can occur in the RET proto-oncogene, which codes for a receptor tyrosine kinase involved in cellular growth and differentiation. These variants can result in a gain of function of the RET protein, promoting tumorigenic processes and leading to a wide spectrum of clinical conditions with incomplete penetrance [5]. The most prevalent is medullary thyroid carcinoma (MTC), which occurs in both syndromic and isolated forms. RET germline variants account for 95-98% of hereditary MTC cases. In this context, it is a clinical feature of multiple endocrine neoplasia type 2 (MEN2). RET variants are also associated with sporadic cases of MTC, accounting for approximately 50% of somatic mutations. Identifying the specific RET variant is critical to diagnosis, prognosis, and response to treatment. In this sense, targeted therapies, like tyrosine kinase inhibitors, can be applied to treat RET-driven tumors [6,7].

The co-occurrence of CD with RET gene variants is extremely rare, with only two cases described in the literature so far, emphasizing the complexity of endocrine pathologies and their pathogenic pathways [8,9]. Cushing disease shares with RET-related conditions a common thread of dysregulation in endocrine tissues. This study, therefore, highlights the importance of genetic testing when faced with multiple extremely rare phenotypes. Understanding the genetic basis of these complex phenotypes can lead to more effective treatments that target the root causes rather than merely addressing the symptoms. Additionally, it can enhance patient care by enabling the prediction and prevention of potentially undiagnosed conditions.

## Case presentation

A 35-year-old male patient was referred to endocrinology outpatient care with a suspicion of Cushing syndrome. He complained about facial plethora, moon face, headache, thinning of the skin, easy bruising, notion of poor skin wound healing, proximal muscle weakness, fatigue, decreased libido, apathy, and anhedonia. The patient’s medical history revealed hypertension, on therapeutic target with enalapril 20 mg per day and lercanidipine 20 mg per day for nine years, bilateral middle carotid artery aneurysms with dysplastic and tortuous features, and intracranial fusiform dolichoectasia mainly in the basilar artery for which he was under follow-up by vascular surgery outpatient care. The patient denied taking any other chronic or current medication. Regarding family medical history, his mother passed away at the age of 61 years due to an aneurysm rupture. His father, aged 65 years, has a history of ischemic cardiomyopathy and type 2 DM. The older brother, aged 42 years, was diagnosed with bladder cancer at 30 years of age, with no recurrence reported since. The younger brother, aged 30 years, has a history of childhood epilepsy and psychiatric pathology. Lastly, the daughter, who is five years old, experienced a stroke in the left cerebral artery at an unknown date and has right upper limb monoparesis with difficulty in fine motor skills. There was no other relevant family medical history.

**Figure 1:**
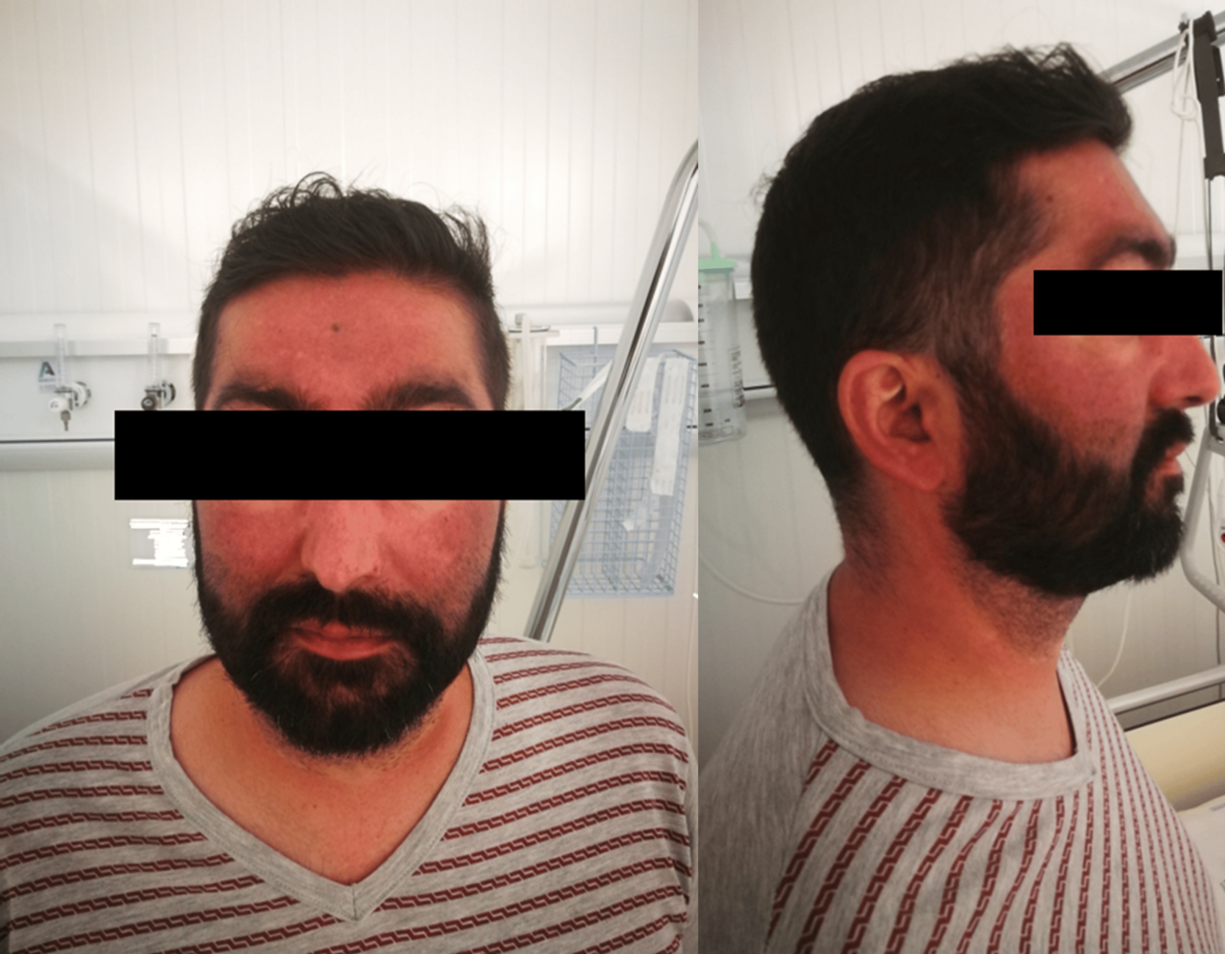
Patient photos on the first physical examination demonstrating a moon face and marked facial plethora.

On physical examination, the patient weighed 71 kg with a height of 175 cm and a body mass index of 23.2 kg/m^2^. His blood pressure was 136/70 mmHg with a heart rate of 66 bpm. Upon physical examination, the patient displayed facial fullness and plethora, supraclavicular fat deposition, abdominal purple striae, muscular atrophy of the lower limbs, and central obesity (abdominal circumference of 97 cm) (Figure 1).

Laboratory investigation revealed augmented late-night salivary cortisol, demonstrating loss of cortisol circadian rhythm; low-dose overnight dexamethasone suppression test indicated failure in suppressing the hypothalamic-pituitary-adrenal axis (HPA); 24-hour urinary free cortisol excretion value indicated elevated levels of circulating free cortisol; plasmatic ACTH was measured twice, with values of 42.8 pg/mL and 37.6 pg/mL, confirming ACTH-dependent Cushing syndrome (Table 1). Of importance in the remaining blood analysis were the findings of hypogonadotropic hypogonadism and prediabetes. The rest of the blood work had no other meaningful variation. A bone densitometry test was performed, revealing a bone mineral density below the expected range for age (Table 1). Afterward, a pituitary MRI was carried out, demonstrating no lesions. A high-dose dexamethasone test was performed (Table 2), suggesting a pituitary ACTH secretion, followed by a BIPSS, revealing a central-to-peripheral ACTH maximal ratio of 25.6 in basal conditions and a maximal ratio of 120.4 at three minutes after corticotropin-releasing hormone (CRH) stimulation, further sustaining the diagnosis of CD (Table 3).

**Table 1:** Biochemical evaluation and bone density test results of the patient on the first evaluation. ACTH: adrenocorticotropic hormone; DEXA: dual-energy X-ray absorptiometry; DM: diabetes mellitus; FSH: follicle-stimulating hormone; HbA1c: glycated hemoglobin; LH: luteinizing hormone; FT4: free thyroxine; TSH: thyroid-stimulating hormone; UFC: urinary free cortisol

Laboratory analysis (reference range)	First evaluation
24 h UFC (4.3-176.0 µg/24 h)	408.0 µg/24 h
Salivary cortisol (0-0.208 µg/dL)	Late-night: 1.10 µg/dL
1 mg overnight dexamethasone suppression test (serum cortisol <1.8 µg/dL)	16.0 µg/dL
ACTH (9-52 pg/mL)	42.8 pg/mL
8 am serum cortisol (6.2-19.4 µg/dL)	22.1 µg/dL
FSH (3.4-4.8 mIU/mL)	4.3 mIU/mL
LH (1.7-8.6 mIU/mL)	3.8 mIU/mL
Total testosterone (2.8-8.0 ng/mL)	2.62 ng/mL
TSH (0.30-3.18 µIU/mL)	0.28 µIU/mL
FT4 (1.01-1.65 ng/dL)	1.24 ng/dL
Prolactin (4.04-15.2 ng/mL)	20.9 ng/mL
HbA1c (5.7-6.4%: prediabetes)	5.9%
Bone density test (DEXA)	Lower spine: Z-score -2.3
	Femoral neck: Z-score -2.2
	Total proximal femur: Z-score -2.2

**Table 2:** High-dose dexamethasone test results during follow-up

High-dose dexamethasone test	Day 1	Day 2
8 am serum cortisol	22.1 µg/dL	3.8 µg/dL

**Table 3:** BIPSS results during follow-up. ACTH: adrenocorticotropic hormone; BIPSS: bilateral inferior petrosal sinus sampling

BIPSS	ACTH peripheral (pg/dL)	ACTH central-left (pg/dL)	ACTH central-right (pg/dL)
0’	27.0	692.0	635.0
3’	32.9	3960.0	3757.0
5’	65.9	4074.0	1758.0
10’	78.0	2031.0	946.0
15’	27.8	779.0	558.0

**Figure 2:**
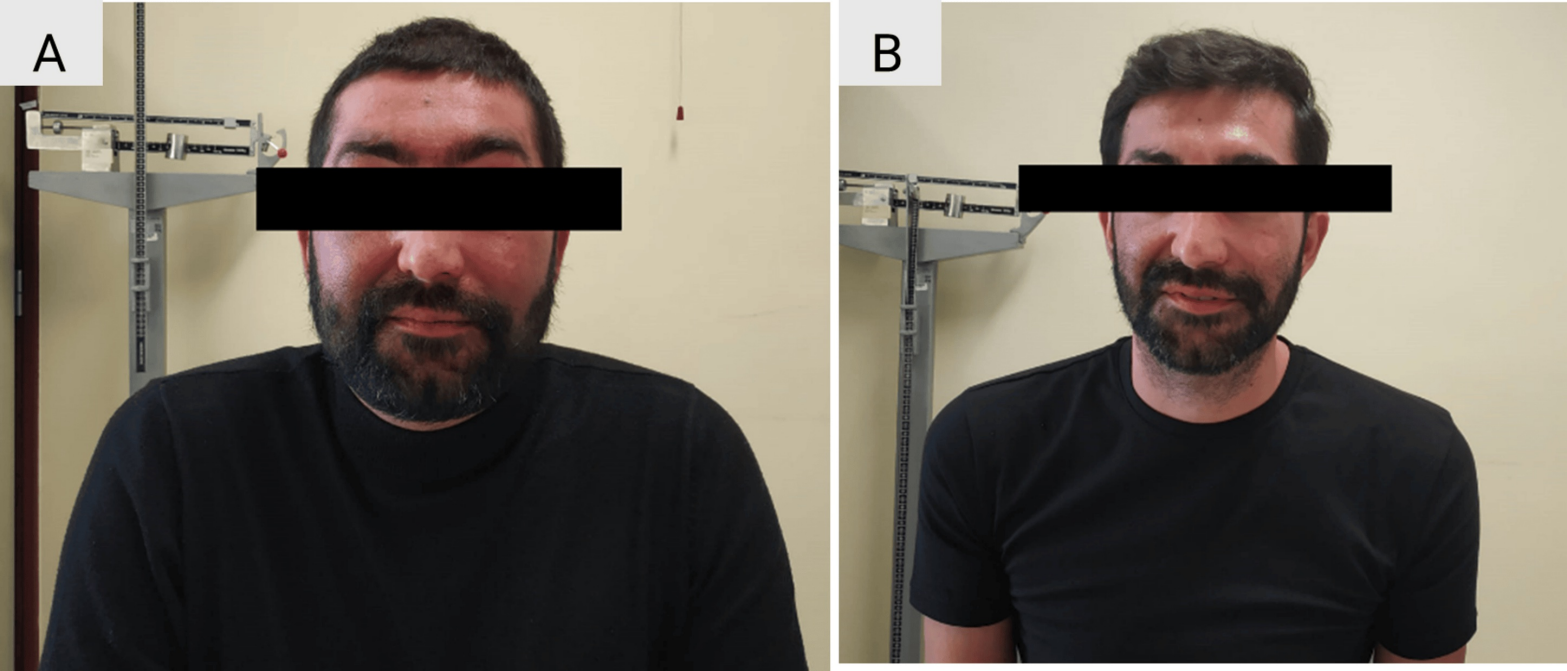
The patient before surgery (A) and a few months after surgery (B) demonstrating a recovery of facial features and loss of weight.

Shortly after, a transsphenoidal endoscopic surgery was performed. There were no major postoperative complications, and histopathological and immunohistochemical analysis of the sample confirmed the presence of a densely granulated corticotropinoma. Surgical success was achieved, as confirmed by a morning serum cortisol level of 1.4 µg/dL, indicating initial remission of CD. The patient started on glucocorticoid replacement therapy with prednisolone 5 mg per day, and instructions were given about stress dosing titration for intercurrent illnesses or surgical procedures. A few months later, the patient displayed biochemical and clinical improvement (Table 4). There was a recovery of facial features, a reduction in abdominal circumference, and a decrease in weight (65 kg) (Figures 2A, 2B). Additionally, his mood improved, and he experienced less fatigue. His arterial blood pressure dropped, and he no longer required antihypertensive medication. Furthermore, there was a complete recovery of his hypogonadotropic hypogonadism and prediabetes. To date, prednisolone has been progressively tapered to a daily dose of 2.5 mg, demonstrating improvement in the HPA axis function (Table 5).

**Table 4:** Biochemical evaluation of the patient after TSS. *Suspension of prednisolone 5 mg on the previous day. FSH: follicle-stimulating hormone; HbA1c: glycated hemoglobin; LH: luteinizing hormone; FT4: free thyroxine; TSH: thyroid-stimulating hormone; TSS: transsphenoidal surgery; UFC: urinary free cortisol

Laboratory analysis (reference range)	After TSS*
24 h UFC (4.3-176.0 µg/24 h)	36.0 µg/24 h
8 am serum cortisol (6.2-19.4 µg/dL)	0.1 µg/dL
FSH (3.4-4.8 mIU/mL)	4.1 mIU/mL
LH (1.7-8.6 mIU/mL)	4.7 mIU/mL
Total testosterone (2.8-8.0 ng/mL)	7.90 ng/mL
TSH (0.30-3.18 µIU/mL)	1.01 µIU/mL
FT4 (1.01-1.65 ng/dL)	1.17 ng/mL
Prolactin (4.04-15.2 ng/mL)	14.9 ng/mL
HbA1c (5.7-6.4%: prediabetes)	5.6%

**Table 5:** Biochemical evaluation of the patient in the last endocrinology outpatient care appointment. *Suspension of prednisolone 2.5 mg on the previous day. FSH: follicle-stimulating hormone; LH: luteinizing hormone; FT4: free thyroxine; TSH: thyroid-stimulating hormone

Laboratory analysis (reference range)	Last evaluation*
Salivary cortisol (0-0.208 µg/dL)	Late night: 0.106 µg/dL
8 am serum cortisol (6.2-19.4 µg/dL)	11.9 µg/dL
FSH (3.4-4.8 mIU/mL)	4.9 mIU/mL
LH (1.7-8.6 mIU/mL)	8.1 mIU/mL
Total testosterone (2.8-8.0 ng/mL)	8.1 ng/mL
TSH (0.30-3.18 µIU/mL)	1.77 µIU/mL
FT4 (1.01-1.65 ng/dL)	1.54 ng/dL
Prolactin (4.04-15.2 ng/mL)	16.7 ng/mL

In the ensuing months, vascular surgery follow-up recommended that the patient undergo corrective surgery for the left carotid aneurysm. The procedure was performed with no significant complications. Due to vascular abnormalities also present in family members, he was referred to a medical genetics consultation for an etiological investigation of a possible genetic connective tissue disorder. In this context, comprehensive genetic testing, including exome sequencing, was performed. Although no connection to a connective tissue disorder was identified, a heterozygous pathogenic variant NM_020975.6:c.2410G>T p.(Val804Leu) in exon 14 of RET gene was detected. Following this discovery, and given that this germline variant carries a moderate risk of aggressive medullary thyroid carcinoma (MTC) and a low likelihood of pheochromocytoma (PHEO) and primary hyperparathyroidism (HPTH), the patient was screened for these conditions by the endocrinology outpatient care team. On physical examination, no notable changes were observed. A thyroid ultrasound was performed, and no suspicious features were observed. Laboratory results showed normal levels of calcitonin, urinary and plasma metanephrines, parathyroid hormone, serum calcium, and serum phosphorus (Table 6).

**Table 6:** Laboratory findings following the identification of the RET variant. FT3: triiodothyronine; FT4: free thyroxine; PTH: parathyroid hormone; TSH: thyroid-stimulating hormone

Laboratory evaluation (reference range)	Value
TSH (0.30-3.18 µIU/mL)	2.08 µIU/mL
FT4 (1.01-1.65 ng/dL)	1.33 ng/dL
FT3 (2.66-4.33 pg/mL)	4.02 pg/mL
Plasma metanephrine (<456.3 pmol/L)	327.0 pmol/L
Plasma normetanephrine (<982.8 pmol/L)	714.0 pmol/L
Calcitonin (0-20 pg/mL)	5.66 pg/mL
PTH (15-65 pg/mL)	54.3 pg/mL
Corrected calcium for serum albumin (2.15-2.50 mmol/L)	2.29 mmol/L
Serum phosphorus (0.87-1.45 mmol/L)	1.17 mmol/L
Serum magnesium (0.60-1.10 ng/mL)	0.86 ng/mL
25-hydroxyvitamin D (50-150 mmol/L)	73.70 mmol/L
Urine metanephrine (264-1729 nmol/dL)	935.0 nmol/dL
Urine normetanephrine (480-2424 nmol/dL)	1243.0 nmol/dL
Urinary 3-methoxytyramine (0-1839 nmol/dL)	1379.0 nmol/dL

**Figure 3:**
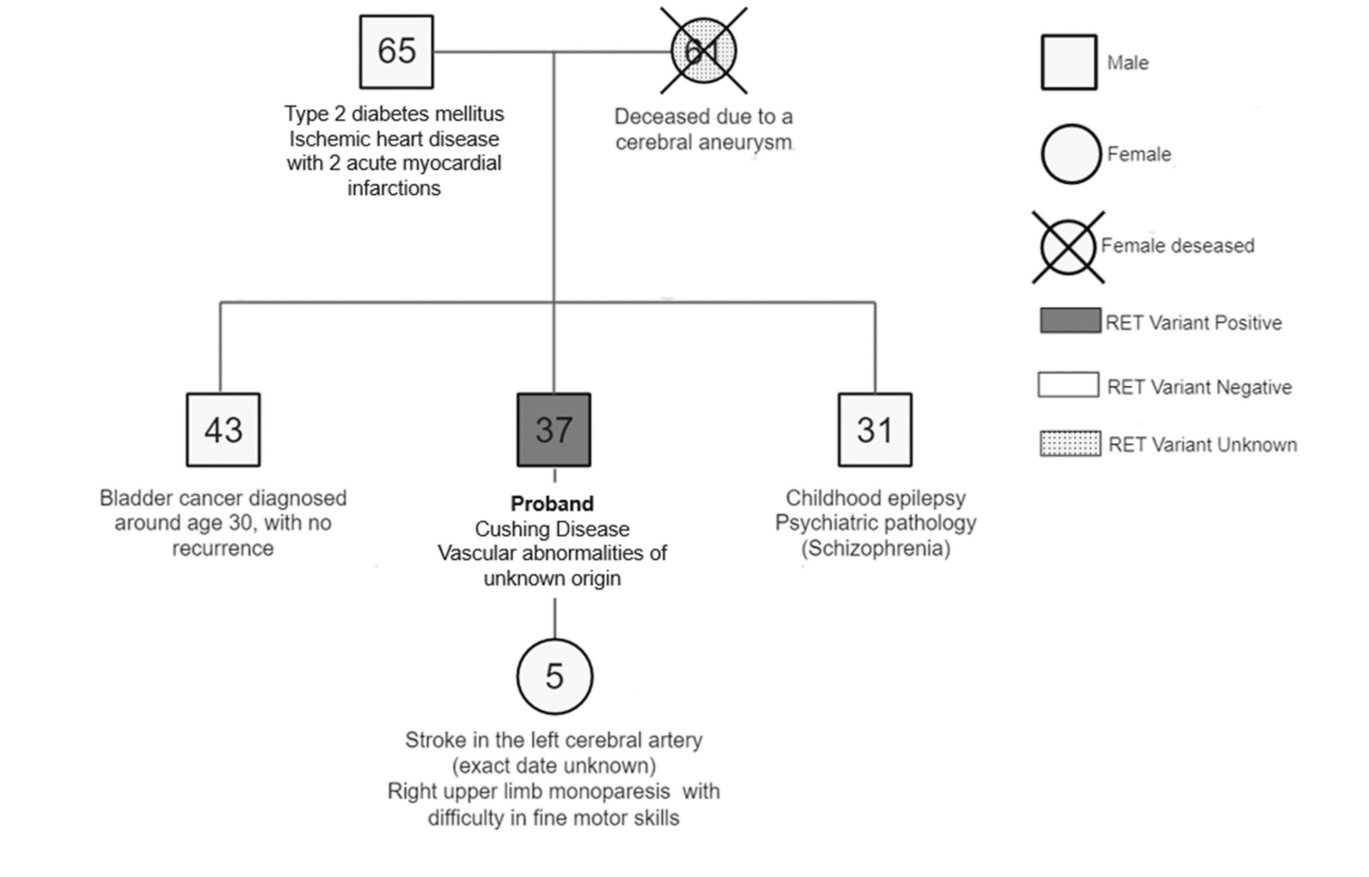
Pedigree of the investigated family regarding RET variant status.

After discussing the prognosis and the potential for the development of MTC, the patient opted for annual surveillance instead of total thyroidectomy. Testing for the familial RET variant was conducted on first-degree relatives, including the father, daughter, and brothers, to assess increased cancer risk and provide genetic counseling regarding reproductive options. All results returned negative (Figure 3).

## Discussion

Tumors causing Cushing disease, corticotropinomas, although less frequent among pituitary tumors (15%), are challenging from an etiopathogenetic point of view due to their sporadic and hereditary occurrence [10]. Also, understanding the distinction between somatic and germline variants is crucial for determining the tumor's origin while also having important implications for treatment and follow-up strategies [11,12].

Corticotropinomas can arise from both somatic and germline variants. Among the somatic variants, the USP8 gene variant is the most common, found in 35-62% of corticotropinomas. This gene’s impairment leads to increased deubiquitination and stabilization of the EGFR, which in turn promotes ACTH secretion. Other, less common somatic variants, such as those affecting the TR4 protein and the BRAF V600E gene variant, also contribute to tumor growth and hormone production through various signaling pathways. Interestingly, using BRAF inhibitor in corticotrophic adenoma cells with a variant in the BRAF V600E gene demonstrated a decrease in ACTH production [4].

Germline variants can also play a central role in the development of corticotropinomas within familial genetic syndromes. The most common is MEN1, with CD occurring in 2-3% of cases. However, the types 2 and 4 syndromes, which can occasionally coexist, are also described in the literature. The AIP gene, while strongly associated with FIPA, is also notably interconnected with the RET proto-oncogene [4]. However, there is currently no evidence to suggest that any variant directly links these two proteins. Despite being well-documented, cases of CD within these syndromes account for less than 5% of all occurrences [13,14].

Conversely, pathogenic variants in the RET gene are associated with MEN2A, MEN2B, familial medullary thyroid carcinoma (MTC), and Hirschsprung disease. MEN2A accounts for approximately 70-80% of MEN2 cases and is primarily characterized by medullary thyroid carcinoma, pheochromocytoma (PHEO), and hyperparathyroidism (HPTH). Certain variants also exhibit well-established genotype-phenotype correlations [15]. Variants in the same codon as our patient - c.2410G>T p.(Val804Leu) - have been shown to be highly variable, even within the same family, with a moderate risk of developing MTC and lower risk for PHEO and HPTH [15,16].

Interestingly, pituitary adenomas are not a classical feature of MEN2, and there have been only four reports in the literature presenting individuals with an association between pituitary adenomas and RET variants, with only two of them bearing both corticotropinomas and RET variants (Table 7). One patient with CD and HPTH developed MTC and bilateral PHEOs almost 20 years later after a clinical diagnosis of MEN1. He underwent genetic testing for selected exons in RET gene, and the variant p.Cys634Arg (c.1900T>C) was reported [8]. Another patient, a 21-year-old male who was diagnosed at the age of 10 years with an ACTH-secreting pituitary microadenoma, at age 16 years developed MTC and was found to have MEN2B with the characteristic M918T RET proto-oncogene variant [9]. While similar phenotypes have been described, the presence of RET variants has not always been convincingly demonstrated [8,17].

**Table 7:** Summary of reported cases of Cushing disease in patients with an identified RET gene variant. *No mention of genetic screening. CD: Cushing disease; M: male; MEN: multiple endocrine neoplasia; MTC: medullary thyroid cancer; PHEO: pheochromocytoma; HPTH: primary hyperparathyroidism; PTH-T: parathyroidectomy; RT: radiotherapy; TSS: transsphenoidal surgery; TT: total thyroidectomy; y/o: years-old

Studies	Age/gender	Clinical manifestations (age of diagnosis)	Treatment	Outcome	Variant of the RET gene identified	Family history
Naziat et al. (2013) [8]	68/M	CD (48 y/o), HPTH (58 y/o), CD recurrence (63 y/o), metastatic MTC (66 y/o), bilateral PHEO (66 y/o), MEN2A (66 y/o)	TSS (2), PTH-T, TT, neck RT	Hypopituitarism	Cys634Arg	Son: HPTH, Cys634Arg RET gene variant, prophylactic TT: MTC
Kasturi et al. (2017) [9]	21/M	CD (10 y/o), metastatic MTC (16 y/o), MEN2B (16 y/o)	TSS (3), left thyroid lobectomy	Hypopituitarism, stable disease course on vandetanib	M918T	No thyroid, parathyroid, pituitary, adrenal, or pancreatic tumors were identified*
Current case 2024	35/M	Vascular abnormalities (33 y/o), CD (35 y/o)	TSS	Surveillance for MTC, HPTH, PHEO, no manifestations to date	Val804Leu	Mother: aneurysm (deceased); daughter with cerebral stroke; father, brothers, and daughter negative for RET variant

Germline RET gene variants are a key factor in MEN2 syndromes. Studies indicate that up to 95% of MEN2A cases and 50% of MEN2B cases are inherited, while approximately 5-9% of MEN2A and 50% of MEN2B cases arise from a de novo germline heterozygous pathogenic variant. These syndromes are inherited in an autosomal dominant manner, meaning that each offspring of an individual with MEN2 has a 50% chance of inheriting the RET pathogenic variant. Once a RET pathogenic variant is identified in a family member, genetic testing for at-risk asymptomatic relatives, as well as prenatal and preimplantation genetic testing, becomes feasible [15]. A genetic study was promptly conducted on the first-degree relatives of our patient. At the time, the patient's mother was deceased, and there was no available sample to test for the RET familial variant. No maternal relatives were available for testing. For this reason, we can’t establish with certainty whether our proband’s variant was inherited or occurred de novo.

Our patient was also being followed for his vascular abnormalities, leading to a genetic investigation of a potential link to a connective tissue disorder. Marfan syndrome, Ehlers-Danlos syndrome, or fibromuscular dysplasia are just a few examples of rare genetic disorders that can lead to elongation, enlargement, and tortuosity of blood vessel walls. It could result in thrombosis, microembolization, and compression of the brainstem, with or without aneurysm formation [18]. Currently, there are no reports in the literature associating RET gene variants with this particular phenotype [15].

Considering this, should all patients with isolated Cushing disease undergo comprehensive genetic testing? Current guidelines generally recommend genetic screening only when there is a strong suspicion of a familial link or when there is an association with another endocrine disorder that raises the possibility of a known syndrome, such as MEN1 and AIP-associated syndromes, which are known to predispose to pituitary tumors [19,20]. A recent consensus statement on genetic screening for CD recommends genetic testing in all familial and/or pediatric forms of Cushing syndrome of adrenal or pituitary origin. We recommend screening for a genetic cause of Cushing syndrome, by gene panel or exome sequencing, in the following: associated clinical manifestations suggestive of a particular genetic syndrome, family history of pituitary adenoma, corticotroph macroadenoma in individuals below 30 years of age at diagnosis, and corticotroph microadenoma in children [20].

However, exceptions may be warranted in cases where patients present with rare comorbidities, as seen in our patient, who, for example, exhibits both CD and personal and familial vascular abnormalities of unknown etiology. Such presentations may indicate the possibility of a pleiotropic disorder, which could justify the need for comprehensive genetic testing to identify a potential underlying genetic etiology.

## Conclusions

The association between a pathogenic variant in RET and Cushing disease is unlikely but should be considered. This emphasizes the importance of genetic testing in diagnosing diseases, particularly when a patient's phenotype presents with multiple rare conditions. In patients with Cushing disease who also exhibit signs of other endocrine conditions, and after excluding MEN1, consideration should be given to the possibility of MEN2. In any case, the discovery of an unexpected germline pathogenic variant in RET implies meticulous follow-up and timely intervention of associated endocrine abnormalities and may also contribute to a better understanding of the genetic interplay in endocrine disorders.

To the best of our knowledge, this study represents the third report of a RET variant associated with Cushing disease. This number could potentially be underrepresented since the majority of CD cases do not undergo genetic testing, and while this rare association can be due to coincidence, we cannot exclude the possibility that they could share a common pathogenic mechanism, emphasizing the need for further studies underlying this condition.
